# Potential Application of Pin-to-Liquid Dielectric Barrier Discharge Structure in Decomposing Aqueous Phosphorus Compounds for Monitoring Water Quality

**DOI:** 10.3390/ma14247559

**Published:** 2021-12-09

**Authors:** Gyu Tae Bae, Jae Young Kim, Do Yeob Kim, Eun Young Jung, Hyo Jun Jang, Choon-Sang Park, Hyeseung Jang, Dong Ho Lee, Hyung-Kun Lee, Heung-Sik Tae

**Affiliations:** 1 School of Electronic and Electrical Engineering, College of IT Engineering, Kyungpook National University, Daegu 41566, Korea; doctor047@knu.ac.kr (G.T.B.); jyk@knu.ac.kr (J.Y.K.); eyjung@knu.ac.kr (E.Y.J.); bs00201@knu.ac.kr (H.J.J.); 2 ICT Creative Research Laboratory, Electronics and Telecommunications Research Institute (ETRI), Daejeon 34129, Korea; nanodykim@etri.re.kr; 3 Department of Electrical and Computer Engineering, College of Engineering, Kansas State University, Manhattan, NY 66506, USA; purplepcs@ksu.edu; 4 School of Electronics Engineering, College of IT Engineering, Kyungpook National University, Daegu 41566, Korea; sophia9733@gmail.com (H.J.); dhlee@ee.knu.ac.kr (D.H.L.)

**Keywords:** atmospheric pressure air plasma, phosphorus compound decomposition, pin-to-liquid dielectric barrier discharge, pin-to-liquid discharge, water quality monitoring

## Abstract

Here, we proposed a pin-to-liquid dielectric barrier discharge (DBD) structure that used a water-containing vessel body as a dielectric barrier for the stable and effective treatment of aqueous solutions in an open atmosphere. To obtain an intense pin-to-liquid alternating current discharge using a dielectric barrier, discharge characteristics, including the area and shape of a ground-plate-type electrode, were investigated after filling the vessel with equivalent amounts of water. Consequently, as the area of the ground electrode increased, the discharge current became stronger, and its timing became faster. Moreover, we proposed that the pin-to-liquid DBD reactor could be used to decompose phosphorus compounds in water in the form of phosphate as a promising pretreatment method for monitoring total phosphorus in water. The decomposition of phosphorus compounds using the pin-to-liquid DBD reactor demonstrated excellent performance—comparable to the thermochemical pretreatment method—which could be a standard pretreatment method for decomposing phosphorus compounds in water.

## 1. Introduction

Atmospheric pressure (AP) plasmas are ionized gaseous collections containing multiple charged particles, excited species, and radicals that are highly chemically reactive but have a very short lifespan [1,2,3,4,5]. These plasmas exhibit non-thermal discharge behaviors due to partial ionization [6,7,8]. Thus, although multiple reactive species enable the effective treatment of materials, the temperature of these plasmas is sufficiently low to avoid thermal damage to the region being treated. This feature has great potential for using AP plasmas in applications that process thermally sensitive materials, including liquid agents [9,10,11,12].

Pin-to-plate discharge is one of the simplest approaches to effectively generate AP plasma in an open atmosphere [13,14,15]. The pin-to-plate electrode configuration allows for facile air discharge without additional gas discharge owing to the pointed end of the pin-shape electrode inducing a localized electric field enhancement. As these devices have a simple structure and are easy to control, multiple modified devices with pin-to-plate electrode configurations, such as pin-to-plate dielectric barrier discharge (DBD) and pin-to-liquid discharge, have been reported [16,17,18,19]. Pin-to-plate DBD, where a dielectric barrier is placed between a pin-type metal electrode and a metal plate electrode, can be affective when a stable plasma process is required at AP with the dielectric barrier properly controlling the discharge duration [16]. For the easy control of the discharge current flow, a metal plate electrode is covered with a dielectric layer of constant thickness, and air plasma is generated by an alternating current (AC) voltage. Pin-to-liquid discharge directly occurs between the pin-type metal electrode and water surface [17,18]. In most cases, a metal plate that serves as the ground (GND) electrode is immersed in a water container to intensify pin-to-liquid discharge [18,19]. In this case, the water placed on the metal plate is treated with air plasma using pin-to-plate discharge.

Currently, our research group is interested in the potential applications of plasma-to-liquid systems for decomposing aqueous phosphorus compounds for monitoring water quality when a considerable amount of phosphorus is present in domestic sewage, factory wastewater, or agricultural water is introduced in a river or lake. Note that eutrophication promotes the proliferation of algal blooms using phosphorus as a nutrient, thereby generating green and red tides [20,21], which can destroy aquatic ecosystems by rapidly reducing the amount of dissolved oxygen in water necessary for aquatic plants and animals to survive. Therefore, research on total phosphorus in water is actively conducted worldwide, and the demand for real-time, on-site monitoring is gradually increasing [22,23].

As phosphorus in aquatic ecosystems exists as various compounds, it is impossible to detect and monitor all the species in water. Thus, the total phosphorus should generally be measured after various phosphorus compounds are decomposed in the form of orthophosphate, PO_4_^3−^, through a pretreatment process. The ascorbic acid method, known as a standard method for examining wastewater, is based on analyzing the phosphorus decomposed from various compounds in water [24,25,26]. For the precise monitoring of total phosphorus in water, pretreatment that effectively decomposes phosphorus compounds into phosphate has become a critical process. A representative pretreatment used for the ascorbic acid method is a thermochemical method using a strong acid-based chemical such as potassium persulfate [27,28]. This method, which oxidatively decomposes phosphorus compounds at high temperatures (>120 °C) and under high-pressure (>1.1 kg cm^−2^) conditions for 30 min, has multiple disadvantages, including long analysis times due to the heating/cooling periods required for high-pressure heating processes, large equipment size, and the use of chemicals as oxidizing agents.

In this study, we propose a pin-to-liquid DBD structure that uses a water-containing vessel body as a dielectric barrier for the stable and effective treatment of aqueous solutions in an open atmosphere. As the water vessel cannot be plate-shaped without a sidewall, the GND electrode can be transformed in a 3D shape that covers not only the outside of the bottom of the container but also that of the sidewall. To obtain an intense pin-to-liquid DBD, discharge characteristics as per the area and shape of the GND electrode are examined after filling the vessel with equivalent amounts of water. In addition, we examine whether the proposed pin-to-liquid DBD effectively decomposes aqueous phosphorus compounds and demonstrate that the proposed plasma pretreatment can be a novel pretreatment approach of the ascorbic acid method that is comparable to conventional thermochemical pretreatment methods.

## 2. Materials and Methods

### 2.1. Atmospheric Pressure Plasma System

**Figure 1:**
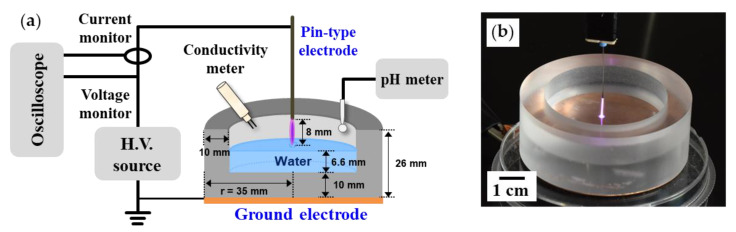
Pin-to-liquid dielectric barrier discharge (DBD) system: (**a**) Schematic of the atmospheric pressure plasma system comprising a pin-to-liquid DBD device, a high-voltage power supply, and measurement instruments, and (**b**) image of the pin-to-liquid DBD structure during air plasma generation.

Figure 1a shows the schematic of the AP plasma system used in this study. A sinusoidal voltage with a peak value of 5 kV and a frequency of 27 kHz was applied to the pin-to-liquid DBD structure using an inverter-type power source. To observe temporal electrical properties during plasma generation, voltage and current waveforms from the powered electrode were monitored using a high-voltage probe (P6015A, Tektronix Inc., Beaverton, OR, USA) and a current transformer (4100, Pearson Electronics Inc., Palo Alto, CA, USA). An intensified charge-coupled device (ICCD) camera (PI-MAX II, Princeton Instruments Inc., Trenton, NJ, USA) equipped with a multispectral imaging lens (UV Nikkor AI-S 105 mm, Nikon Corp., Tokyo, Japan) was used in the shutter mode to identify the spatial distribution of the glow emission of the air plasma. A fiber optic spectrometer (USB-2000+, Ocean Optics Inc., Dunedin, FL, USA) was employed to identify reactive species generated using the pin-to-liquid discharge.

### 2.2. Pin-to-Liquid Dielectric Barrier Discharge Structure

Figure 1b shows schematics and images of the pin-to-liquid DBD device with the water vessel with a Cu plate covering the entire bottom surface at AP. To clearly observe the optical behavior of the air plasma, the water vessel was fabricated as a cylindrical container made of translucent polycarbonate with a dielectric constant (ε_r_) of 2.9. The inner and outer diameters of cylindrical vessel were 25 and 35 mm, respectively, and the height was 26 mm. The thicknesses of the bottom and sidewall were both 10 mm. A tungsten wire with a diameter of 500 μm was used as the pin-type power electrode. When 13 mL of water was added to this vessel, the height of the water surface was 6.6 mm. The air spacing between the pin-type electrode and water surface was fixed at 8 mm. As the plate-shaped electrode could be attached to the outside of the water vessel, it could extend to both the sidewall and bottom of the vessel. The pin-to-liquid DBD devices using different areas and GND-electrode shapes and their discharge characteristics are investigated in detail in Section 3.2.

### 2.3. Preparation of Phosphorus Compound and Its Decomposition Using Pin-to-Liquid DBD

Note that 9.9 mg of β-glycerol phosphate disodium salt pentahydrate (BGP; C_3_H_7_Na_2_O_6_P·5H_2_O, Sigma-Aldrich Inc., St. Louis, MO, USA) was dissolved in 1 L of deionized (DI) water to prepare a BGP solution with a phosphorus concentration of 1.0 mg/L. This prepared BGP solution was dispensed (13 mL) into the water vessel of the pin-to-liquid DBD reactor and irradiated with air plasma for 10 min, as shown in Figure 1b. The orthophosphate concentration in the BGP solution before and after plasma treatment was determined by ion chromatography equipment (ICS3000, Dionex Corp., Sunnyvale, CA, USA) at the Korea Institute of Basic Science (KBSI, Busan, Korea). The volume of the injection loop was 20 μL and the column temperature was 30 °C.

### 2.4. Thermochemical Method for Decomposition of Phosphorus Compound

Note that 0.4 g of potassium persulfate (K_2_S_2_O_8_, Sigma-Aldrich Inc., St. Louis, MO, USA) was dissolved in 10 mL of DI water to prepare a 4% potassium persulfate solution. Fifty mL of the BGP solution prepared in Section 2.2 and 10 mL of the 4% potassium persulfate solution were mixed, placed in a PTFE bottle, sealed, heated at 120 °C for 30 min, and then cooled to room temperature.

### 2.5. Ascorbic Acid Reduction Method

The ascorbic acid reduction method—the most extensively used colorimetric method for aqueous phosphorus analysis—was performed as follows [26]. First, a phosphate standard solution with a phosphorus concentration of 1.0 mg/L was prepared by dissolving 4.4 mg of potassium phosphate monobasic (KH_2_PO_4_, Sigma-Aldrich Inc., St. Louis, MO, USA) in 1 L of DI water. Then, the 1.0 mg/L standard solution was diluted to prepare standard solutions containing 0.2, 0.4, 0.6, and 0.8 mg/L of phosphorus. Furthermore, 10 mL of these standard solutions were mixed with 0.18 g of the colorimetric reagent for the ascorbic acid method (HI736, Hanna Instruments Inc., Woonsocket, RI, USA) and reacted for 10 min. Visible-NIR absorption spectra of the prepared standard solutions were measured using a miniature optic spectrometer (USB-4000, Ocean Optics Inc., Dunedin, FL, USA). A calibration curve was obtained from the absorbance of the phosphate standard solutions at 710 nm [29]. The decomposition of BGP solution in orthophosphate (PO_4_^3−^) by plasma and thermochemical pretreatments was examined by comparing the calibration curves obtained from the phosphate standard solutions.

### 2.6. Statistical Analysis

All quantitative data are presented as the mean ± standard deviation. Measurements were performed on three replicates for each experiment (n = 3). The decomposition efficiency to phosphate form was statistically analyzed by unpaired Student’s t-test to compare the differences between the means using the least significant differences at *p* < 0.05, *p* < 0.01, and *p* < 0.001.

## 3. Results and Discussion

### 3.1. Discharge Characteristics of Pin-to-Liquid Dielectric Barrier Discharge

The fabricated vessel of the pin-to-liquid DBD device not only served as a water container but also a dielectric barrier that temporarily controlled the discharge current. The use of a dielectric barrier allowed for generating stable glow plasmas without a ballast resistor in the plasma driving circuit. The stability and temporal electrical properties of the generated plasma were investigated by monitoring the applied voltage and current waveforms during air plasma generation.

**Figure 2:**
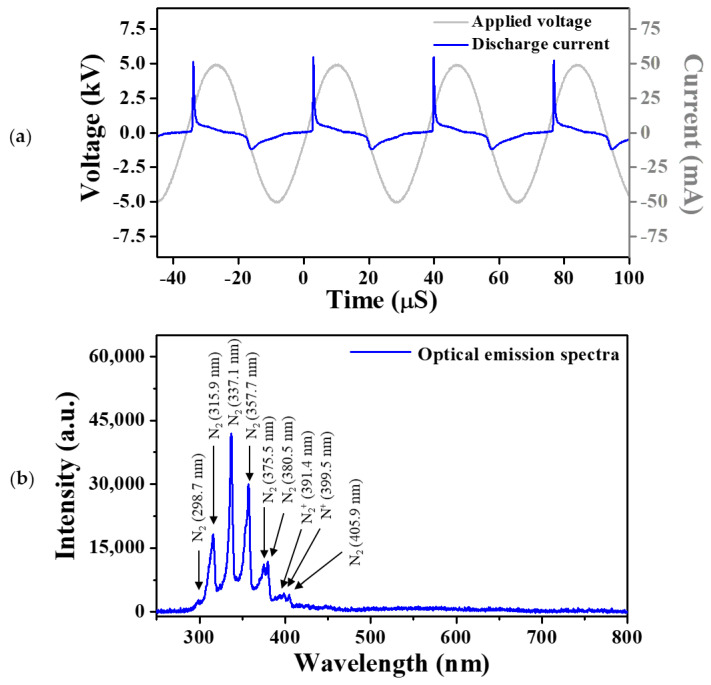
(**a**) Temporal distribution of applied voltage and discharge current during air plasma generation and (**b**) optical emission spectrum measured during pin-to-liquid DBD.

Figure 2a shows the discharge current waveforms during four cycles of a sinusoidal applied voltage. The discharge current was obtained by excluding the displacement current from the total measured current. The discharge current waveform demonstrated that each discharge occurred during the rising and falling periods of the voltage waveform, which indicated that discharges were stably produced even when the pin-type electrode served as an anode and a cathode. Considering that the intensity of the discharge current generated in each cycle was constant, it was confirmed that the pin-to-liquid DBD system was very stable. When the pin-type electrode served as an anode, the intensity of the discharge current peak was approximately five-times higher than when it served as a cathode, which was attributed to the different electrode shapes. At the pointed end of the pin-type electrode, discharge occurred more readily due to the localized electric field enhancement; thus, the discharge intensity generated when the pin-type electrode acted as an anode was considerably stronger.

To identify the reactive species generated by the pin-to-liquid DBD in ambient air, the emission spectra of a plasma plume were monitored using the fiber optic spectrometer. Figure 2b shows the emission spectra from 250 to 800 nm, indicating that excited N_2_, N_2_^+^, and N^+^ existed in the plasma plume. As this AP-plasma system did not use discharge gases such as Ar or He, the several excited nitrogen species were primarily from the atmosphere.

### 3.2. Changes in Plasma Intensity According to Area of Plate-Shaped Electrode

**Figure 3:**
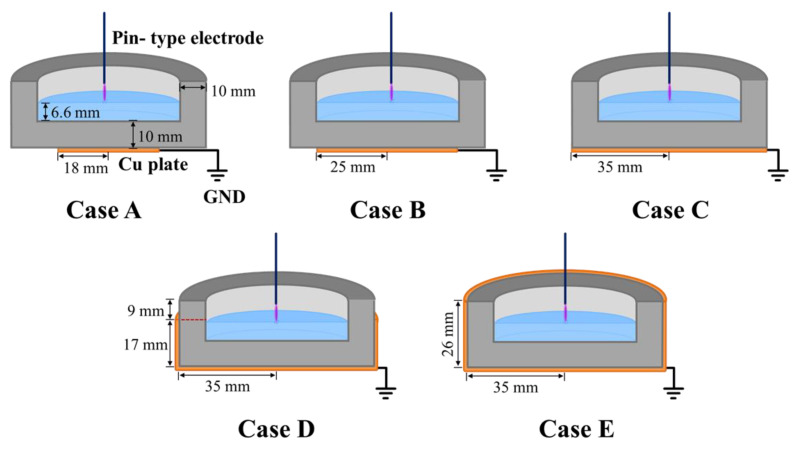
Schematics of the proposed pin-to-liquid DBD structures with GND plate electrodes with five different areas and shapes.

In the proposed pin-to-liquid DBD structure, the metal plate attached to the outside of the bottom of the water vessel served as the counter electrode for the powered electrode and was electrically the GND electrode. As the plate-shaped GND electrode could be attached to the water vessel in the form of enveloping its outer surface, the electrode could be extended to both the sidewall and bottom of the container. Therefore, as shown in Figure 3, the discharge characteristics of the proposed pin-to-liquid DBD structure using five different areas and GND-electrode shapes were investigated. Table 1 presents a description of the GND electrode for each case.

**Figure 4:**
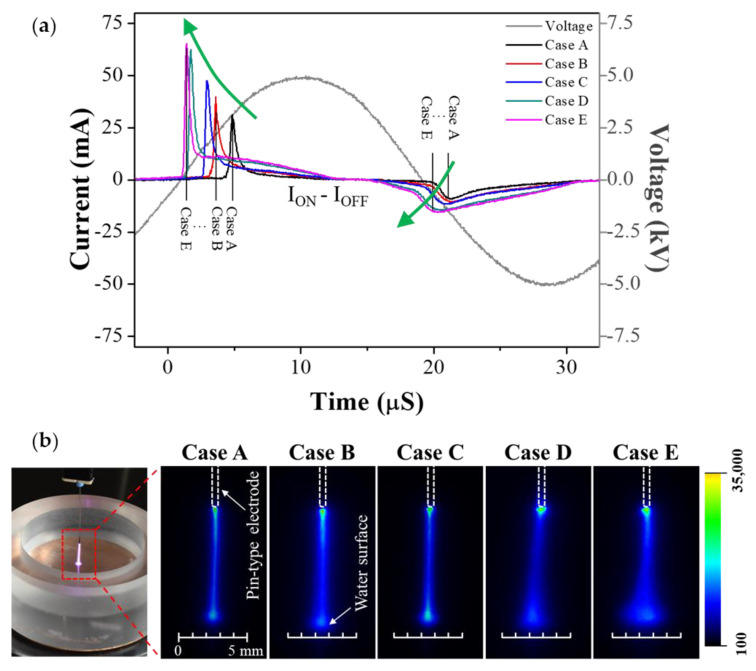
(**a**) Temporal behaviors of discharge current waveform according to the changes in area and shape of plate electrode and (**b**) spatial distribution of the plasma emission in Cases A–E.

Figure 4a shows the discharge current waveform vs. GND-electrode area when a voltage of 5 kV was applied. A larger GND-electrode area resulted in a stronger discharge current and faster discharge timing. These observations reported that the area of the counter electrode, which was electrically 0 V and not the power electrode to which the high voltage was directly applied, can influence discharge characteristics. Furthermore, the amplitude of the discharge current peak increased not only in the positive half cycle, but also in the negative half cycle of the sinusoidal voltage waveform, which indicated that the plasma became stronger not only when the pin-type electrode served as an anode but also when it served as a cathode. This clearly reported that the residual charged particles generated by the previous discharge accumulated on the dielectric barrier and affected the next alternating discharge. For Cases D and E, where the GND electrode covered the entire bottom surface and further extended to the sidewall, the plasma intensities were much stronger.

Generally, in DBD structures, an increase in the area of the electrode with the dielectric barrier induced a considerable number of residual charged particles accumulated on the dielectric barrier, leading to a stronger AC discharge, even when the voltage applied from the power source remained constant. Therefore, as the area of the GND electrode increased in the proposed pin-to-liquid DBD structure, the effective area of the dielectric barrier increased. This indicates that, even if the applied voltage does not change, the effective electric field can be changed by accumulated charged particles depending on the area and shape of plate electrode, which is an irreplaceable advantage of DBD comparable to the current control characteristics.

To investigate the spatial behavior of pin-to-liquid DBD according to the area of the GND electrode, the generated plasma plume was observed via ICCD imaging measurements. Figure 4b shows emission images of the glow plasma generated between the pin-type electrode and the water surface at a distance of 8 mm, which was measured using the shutter mode of the ICCD camera in Cases A–E. In the ICCD images, the emission intensity of the glow plasma is shown in pseudo-color, and the scale bar represents the emission intensity counts. When air plasma occurred between the pin-type electrode and the water surface in Case A, where the GND electrode was located only at the center of the bottom of the vessel, the generated plasma pulse was narrow, as the electric field was mainly directed downward. As the area of the GND electrode increased, the width of the plasma plume in contact with the water surface increased due to the wider electric-field distribution. The broadest plasma plume was observed in Case E, where the GND electrode covered the sidewall as well as the bottom of the vessel. Case E had a container-shaped GND electrode configuration in which a metal plate surrounded a pin-type power electrode in 3D space. Thus, the direction of the electric field at the end of the pin-type electrode was significantly broadened not only downward but also laterally so that the plasma plume touching the water surface was observed to be significantly wider.

**Figure 5:**
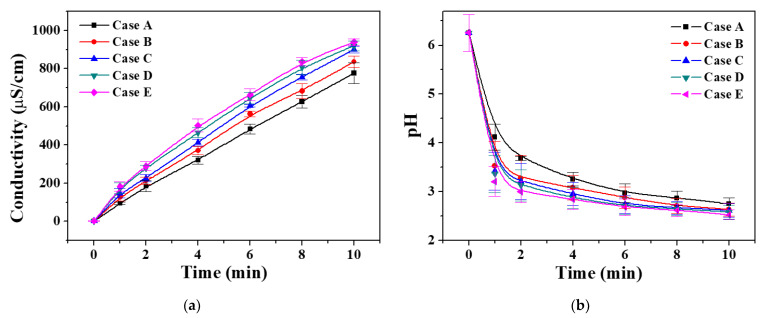
Temporal changes in electrical conductivity (**a**) and pH value (**b**) of DI water treated by pin-to-liquid DBD. All data are presented as the mean ± SD of three repeated experiments.

Figure 5 shows the temporal changes in electrical conductivity and pH during plasma treatment of 13 mL of DI water with the proposed pin-to-liquid DBD reactor. The conductivity of the DI water gradually increased as the plasma treatment time increased and, when it was treated for 10 min, the electrical conductivity increased from 1.9 to 780–950 μS/cm in Cases A–E (Figure 5a). From the comparison results of all five cases (Cases A–E) treated with air plasma for a specific duration, Case E, which obtained the strongest plasma intensity, produced plasma-treated DI water with the highest conductivity. Regarding hydrogen-ion concentration, in all cases, the pH values rapidly decreased and became acidic during the initial 2 min of treatment, indicating that H^+^ was generated quite rapidly from the water molecules by the air plasma (Figure 5b). The pH values decreased rapidly from 6.2 to 3.3–3.7 during the initial 2 min of treatment and then decreased relatively slowly from 3.3–3.7 to 2.6–2.8 for 8 min thereafter. In addition, a higher plasma intensity due to the larger area of the GND electrode meant more hydrogen ions in the plasma-treated DI water were generated.

### 3.3. Decomposition of Phosphorus Compounds in Water by Pin-to-Liquid DBD

To develop a facile pretreatment method suitable for monitoring total phosphorus in water, we measured phosphate production by decomposing phosphorus compounds using the proposed pin-to-liquid DBD structure. Thirteen mL of a BGP solution containing 1.0 mg/L of phosphorus was selected as an example and treated by air plasma for 10 min using the pin-to-liquid DBD reactor with various GND-electrode areas.

**Figure 6:**
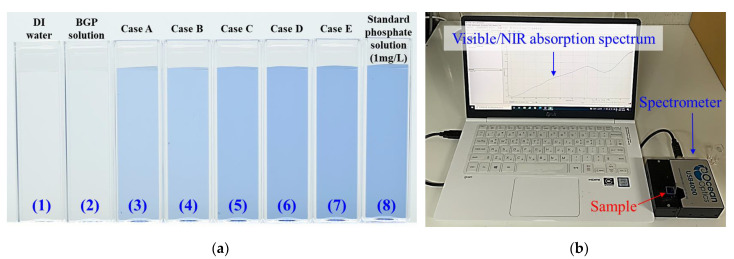
(**a**) Photograph of (1) DI water, (2) untreated BGP solution, (3–7) plasma-treated BGP solutions (Cases A–E), and (8) standard phosphate solution with varying intensities of blue color induced by molybdic acid in the ascorbic acid reduction process for detecting phosphate in water and (**b**) experimental setup for measuring visible-NIR absorption spectra of BGP solution treated by air plasma.

Samples (3–7) in Figure 6a show different blue regions with varying brightness of the plasma-treated BGP solutions after the addition of the colorimetric reagent for detecting and quantifying the phosphate levels in the samples treated by air plasma. The difference in the blue regions was induced by molybdic acid during the ascorbicacid reduction process for detecting phosphate in water. Samples (1) and (2), which were DI water and BGP solutions, contained no phosphate components, which confirmed that the liquids could be transparent even after the addition of a colorimetric reagent. The decomposition levels of the phosphorus compound (BGP) in the form of C_3_H_7_Na_2_O_6_P·5H_2_O into the form of PO_4_^3−^ can be identified by the brightness of the developed blue region, and its brightness deepens from Case A to Case E. It was noticed that the dark blue levels in Cases D and E were not significantly different from that of the phosphate standard solution made with potassium phosphate monobasic (KH_2_PO_4_), as seen in sample (8) in Figure 6a. It is possible to quantify the decomposition-to-phosphate levels of the plasma-treated samples by comparing their absorption spectra at a wavelength of 710 nm with the calibration curve obtained from the phosphate standard solutions containing 0.2, 0.4, 0.6, 0.8, and 1.0 mg/L of phosphorus, as shown in Figure 6b.

**Figure 7:**
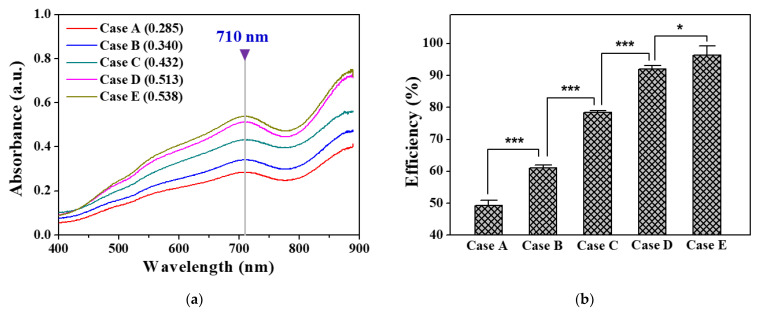
(**a**) Visible-NIR absorption spectrum curves of BGP solution treated by pin-to-liquid DBD reactor with five different GND-electrode areas for 10 min and (**b**) efficiency of phosphorus decomposition to phosphate form by plasma pretreatment. Phosphorus decomposition efficiencies are presented as the mean ± SD of three repeated experiments. Asterisks indicated *p* values. *, *p* < 0.05; ***, *p* < 0.001 by unpaired Student’s *t*-test results.

Figure 7a shows the absorption spectra in the range of 400–900 nm obtained from samples treated by the pin-to-liquid DBD reactor with five different GND-electrode areas. In general, in the ascorbic acid reduction method, the phosphate concentration is quantified by comparing the absorbance at a wavelength of 880 or 710 nm [29]. In this study, only the sample absorbance obtained at a wavelength of 710 nm was used for quantification due to a noise signal at 880 nm. Figure 7b shows the resulting decomposition efficiency of BGP solution into phosphate by plasma pretreatment in Cases A to E. As expected from the results of the darkness level of the blue region (Figure 6a), the phosphorus-decomposition-to-phosphate efficiencies in Cases D and E were as high as 93% and 98%, respectively, confirming that most of the phosphorus compounds were decomposed into phosphate by the pin-to-liquid DBD treatment.

**Figure 8:**
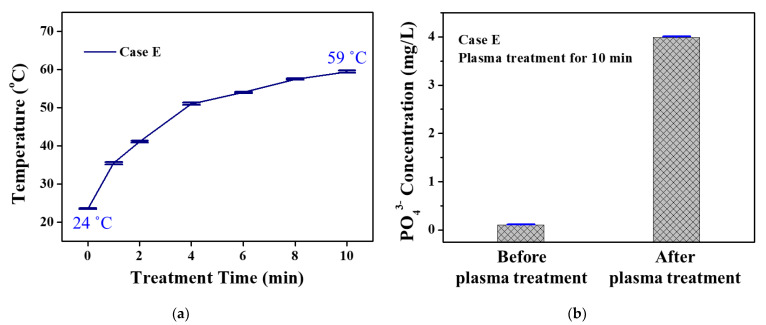
(**a**) Temperature change of BGP solution treated by pin-to-liquid DBD for Case E and (**b**) changes in orthophosphate concentration before and after plasma treatment for Case E in BGP solution via ion chromatography. Data are presented as the mean ± SD of three repeated experiments.

Figure 8a shows the temperature change of the BGP solution by plasma pretreatment for Case E, which had the highest phosphorus decomposition efficiency. Even if the BGP solution was plasma-treated for 10 min for Case E, the solution temperature was lower than 60 °C, which was not high enough to initiate the thermal reaction. Nevertheless, the concentration of orthophosphate in the BGP solution was significantly increased after 10 min of plasma treatment (Figure 8b). This indicated that the plasma treatment was excellent for decomposing BGP in the form of C_3_H_7_Na_2_O_6_P·5H_2_O into orthophosphate in the form of PO_4_^3−^.

**Figure 9:**
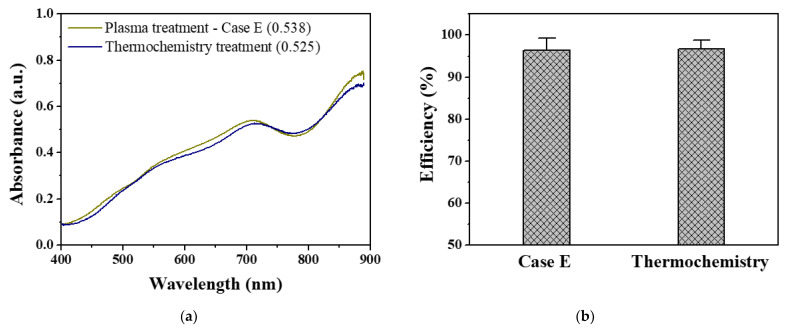
(**a**) Visible-NIR absorption spectra of BGP solution treated by pin-to-liquid DBD (Case E) and thermochemical treatment and (**b**) resulting decomposition efficiency to phosphate form (mean ± SD of three repeated experiments).

Figure 9a shows the absorption spectra of the BGP solution treated with the strongest pin-to-liquid DBD (Case E) and that thermochemically treated with 4% potassium persulfate solution. Using the absorption spectra obtained in Figure 9a, the decomposition efficiency of both pretreatments was determined by comparing their absorbance at a wavelength of 710 nm with that from the 1.0 mg/L phosphate standard solutions (Figure 9b). BGP solutions treated with the oxidizing agent at high temperatures and under high-pressure conditions and those treated by the proposed pin-to-liquid DBD structure were successfully decomposed into phosphate with an efficiency of more than 95%. As a result of unpaired Student’s t-test for the data in Figure 9b, the *p* value was more than 0.1, indicating that there was no significant difference between plasma pretreatment and thermochemical pretreatment in decomposition efficiency. It was confirmed that the proposed plasma method can excellently replace the thermochemical method, which is the current standard method. Therefore, the decomposition approach by air plasma can be directly used as a promising pretreatment process for the ascorbic acid method to determine total phosphorus in water, which is a key indicator of water quality in rivers and lakes. In addition, this pretreatment method using air plasma has the advantages of being environmentally friendly, having a fast analysis duration, and miniaturizing the entire system, making it a more suitable solution for real-time, on-site monitoring of total phosphorus in water.

## 4. Conclusions

In this study, we proposed a pin-to-liquid DBD structure using a water container as a dielectric barrier for the stable and effective treatment of solutions and investigated its electrical and optical properties according to the changes in the area of the GND plate electrode. As the area of the GND electrode increased and took on a three-dimensional shape, the intensity of the discharge current increased, the discharge timing was accelerated, and the plasma plume touching the water surface was widened. We also demonstrated that the proposed pin-to-liquid DBD structure could be used to decompose phosphorus compounds in water into their phosphate form. To develop a facile pretreatment method suitable for monitoring total phosphorus in water, it is also necessary to test whether various organic and inorganic phosphorus compounds can be decomposed by plasma and whether actual phosphorus compounds in raw water of rivers or lakes can be decomposed and detected by plasma. Furthermore, an engineering effort is required to manufacture the entire total phosphorus monitoring system into a single module.

## Figures and Tables

**Table 1:** Summary of descriptions of the five different plate electrodes for each case.

Copper Plate (GND Electrode)	Case A	Case B	Case C	Case D	Case E
**Position**	Bottom	Bottom	Bottom	Bottom + Sidewall	Bottom + Sidewall
**Shape**	Circular plate	Circular plate	Circular plate	Cylindrical container	Cylindrical container
**Area**	10.18 cm^2^	19.64 cm^2^	38.48 cm^2^	75.87 cm^2^	95.63 cm^2^
**Relative area ^1^**	0.26	0.51	1	1.97	2.49

^1^ Ratio of the area of the copper plate (GND electrode) to the bottom area of the water vessel.

## Data Availability

Not applicable.
